# Advancing the application of systems thinking in health: sustainability evaluation as learning and sense-making in a complex urban health system in Northern Bangladesh

**DOI:** 10.1186/1478-4505-12-45

**Published:** 2014-08-26

**Authors:** Eric G Sarriot, Michelle Kouletio, Dr Shamim Jahan, Izaz Rasul, AKM Musha

**Affiliations:** Director, ICF International Center for Design and Research in Sustainable Health and Human Development (CEDARS), 530 Gaither Road Suite 500, Rockville, MD 20850 USA; Health Consultant, US Embassy, 01 BP 2012, Cotonou, Republic of Benin; Country Director, University of Chicago Research, House 4 Road 2B, Sector 4, Uttara, Dhaka 123 Bangladesh; Director of Health Programs, Concern Worldwide, House 15 SW(D), Road 7, Gulshan 1, Dhaka Bangladesh; Country Director, Concern Worldwide, House 15 SW(D), Road 7, Gulshan 1, Dhaka Bangladesh

**Keywords:** Complex adaptive systems, Equity, Evaluation, Health systems, Learning, Participatory, Prevention, Sustainability, Systems thinking, Urban health

## Abstract

**Background:**

Starting in 1999, Concern Worldwide Inc. (Concern) worked with two Bangladeshi municipal health departments to support delivery of maternal and child health preventive services. A mid-term evaluation identified sustainability challenges. Concern relied on systems thinking implicitly to re-prioritize sustainability, but stakeholders also required a method, an explicit set of processes, to guide their decisions and choices during and after the project.

**Methods:**

Concern chose the Sustainability Framework method to generate creative thinking from stakeholders, create a common vision, and monitor progress. The Framework is based on participatory and iterative steps: defining (mapping) the local system and articulating a long-term vision, describing scenarios for achieving the vision, defining the elements of the model, and selecting corresponding indicators, setting and executing an assessment plan,, and repeated stakeholder engagement in analysis and decisions . Formal assessments took place up to 5 years post-project (2009).

**Results:**

Strategic choices for the project were guided by articulating a collective vision for sustainable health, mapping the system of actors required to effect and sustain change, and defining different components of analysis. Municipal authorities oriented health teams toward equity-oriented service delivery efforts, strengthening of the functionality of Ward Health Committees, resource leveraging between municipalities and the Ministry of Health, and mitigation of contextual risks. Regular reference to a vision (and set of metrics (population health, organizational and community capacity) mitigated political factors. Key structures and processes were maintained following elections and political changes. Post-project achievements included the maintenance or improvement 5 years post-project (2009) in 9 of the 11 health indicator gains realized during the project (1999–2004). Some elements of performance and capacity weakened, but reductions in the equity gap achieved during the project were largely maintained post-project.

**Conclusions:**

Sustainability is dynamic and results from local systems processes, which can be strengthened through both implicit and explicit systems thinking steps applied with constancy of purpose.

## Background

Whether we understand the social world (including health systems) as operating through systems, or we take systems thinking simply as a useful mental construct to deal with the complexities of our social world [1], there is a growing attention to systems thinking in multiple fields of development practice and research, including global health. In the global development literature, one may argue that this school of thought started with Amartya Sen’s book, *Development as Freedom* [2], and Rihani’s *Complex Systems: Theory and Development Practice* [3]. Studies and implementation guidelines for ‘systems thinking’ have been published in journal supplements in 2006 and 2007 [4, 5]. The Alliance for Health Policy and Systems Research published *Systems Thinking for Health Systems Strengthening* in 2009 [6], and then supported the publication of a Journal supplement in *Health Policy and Planning* in 2012, dedicated to the applicability of systems thinking tools for health systems strengthening [7]. Over the same period (2008 to 2010), different authors have used case studies to describe how complexity and adaptation play a central part in capacity building [8–10]. Overlapping with the field of global health and global development, the world of evaluation is itself trying to better approach non-linear realities, complexity, and systems thinking [11]. Williams [12] presents 11 evaluation case studies reporting the use of systems methods and concepts to evaluation, and Patton [13] writes and teaches on Developmental Evaluation, a methodology explicitly anchored in an understanding of the world as composed of overlapping open systems. Discussions, research, and evaluation on sustainability in health programs appear to have followed a similar evolution (see “A systems understanding of sustainability” below). Over the same decade and a half, a group of practitioners, working in community health at local levels and globally, followed an interesting if not identical intellectual trajectory, when dealing with the specific issue of improving the sustainability of their interventions. Non-Governmental Organizations (NGOs) working globally in maternal, reproductive, and child health formed the Child Survival Collaborations and Resources Group in 1997 (now known as the CORE Group^a^). In 2000, CORE partnered with a US Agency for International Development (USAID) project implemented by Macro International in a study called the ‘Sustainability Initiative’, in order to improve the conceptualization and implementation of more sustainable strategies in community health [14, 15]. By 2002, this collaboration had produced a tool (the Sustainability Framework) for sustainability planning and evaluation. By this time, Concern Worldwide Inc. (Concern) had been implementing an urban health project in two Municipalities of Northern Bangladesh since 1999. Concern’s interest in capacity building and sustainability led to a time of critical questioning at the time of its mid-term evaluation in 2002, 2 years before the end of the project [16]. Concern needed an evaluation and learning tool able to guide the implementation of project strategies (by the municipalities themselves) toward greater odds of sustainability. The interest in learning, however, bridged the local Bangladeshi and the global context when USAID provided additional funds to Concern to carry out a post-project evaluation 3 and 5 years after the end of the project (2007 and 2009).

### A systems understanding of sustainability

Conceptual and methodological debates about sustainability continue in the literature [17, 18], but the identification of complex systems behaviors as fundamental determinants of sustainability, which was already identified to some extent by a number of past authors [19–21], has become more explicit in recent publications [3, 9, 22–25].

Under the Sustainability Framework, sustainability is seen as resulting from processes taking place in a local system where a wide array of stakeholders share responsibility to generate and maintain positive health outcomes for their community, inclusive of its most vulnerable groups. It offers an interactive model for assessing progress on critical dimensions [26], such as the health outcomes being promoted, characteristics of health services (quality, accessibility, equity), institutional capacity and viability of local government and civil society agencies with long-term responsibility for the outcomes, capacity in beneficiary communities (e.g., social capital, community organization, knowledge/skills, resource mobilization), and socio-ecological conditions enabling the work of these local agents.

This paper presents how Concern adapted and used a systems approach to place sustainability at the front end of project implementation and learning, to build consensus, find common values, use data for learning and adaptive management, and assess progress toward sustainability, during and after the life of the project.

### Urban health in Bangladesh and Concern’s urban health model in Saidpur and Parbatipur (1999–2004)

Bangladesh is a low-income country with poor health indicators. Its under-five mortality decreased rapidly in the 1990s, then slower in the 2000s [27, 28]. The infant mortality rate decreased from 72/1,000 live births in 2004 to 57/1,000 in 2007 according to the Demographic and Health Survey [29]. The fastest growing sector of the population lives in urban areas and a third of those, in urban slums. The urban population grew from 23% of the total population in 2001 [30] to 28% by 2010 [31]. This population is largely vulnerable, impoverished, malnourished, and receives poor health care services [32].

Municipalities are legally tasked with ensuring the delivery of primary health care services to the population but had developed almost no capacity to do so at the onset of Concern’s project. For instance, due to limited resources, public-sector health services were not able to meet the existing needs in 1999. Private health care providers were the main source of curative care, including tertiary and specialized services to the urban populations, but had limited or no interest in providing preventative and health promotion services.

In 1995, the Ministry of Local Government, Rural Development and Cooperatives issued a directive for the effective implementation of expanded programs on immunizations, along with primary health care and family planning services through a coordinated effort involving the Ministry of Health and Family Welfare, NGOs, and private providers. Committees were recommended, although not established, at three different levels to ensure effective health service delivery: inter-ministerial committees, central committees at municipal levels, and Ward Health Committees (WHCs) at the community level.

Concern initiated a USAID-funded child survival project in the municipalities of Saidpur and Parbatipur in Nilphamari and Dinajpur districts in 1998, with full implementation from 1999 to 2004. The two municipalities had a direct beneficiary population of 74,000 women of reproductive age and children under 5. Concern selected what, at the time, was a non-traditional capacity building approach, based on a partnership with the two mayor’s office and their under-resourced municipal health departments (MHDs) [33]. Concern supported the organizational development of the municipality cabinets and through them developed the capacity of WHCs at the community level. In turn, and collaboratively with the MHDs, the WHCs recruited, trained and supported a network of community health volunteers (CHVs) and traditional birth attendants, who carried out community and household-level health promotional activities.

Two years into the project, the results of the mid-term evaluation were very promising but pointed out to the lack of measurable results and signaled important sustainability challenges. Concern’s strategic response to the mid-term evaluation implicitly relied on systems thinking (Table 1). The project, however, also needed a method, an explicit set of processes, to guide a diversity of stakeholders in the pursuit of sustainable health goals. Concern chose the Sustainability Framework as the tool through which it could organize the creative thinking of multiple stakeholders, create a common end-goal, and monitor progress on its redesigned sustainability strategy.

**Table 1:** **Implicit and explicit operationalization of systems thinking for Concern, Saidpur and Parbatipur**

Principle in systems thinking^a^	Concern’s management decision (implicit system approach)	Sustainability Framework (explicit method)
Consider boundaries of system, nested and overlapping systems	• Making the distinction between the national health system and local urban administrative systems, and focusing capacity building on the MHDs	• Local system and stakeholder mapping
		• Identification of interrelated roles between MOHFW and MHD
Construct reality through multiple perspectives	• Voices from all segments of society included in process	• Visioning and scenario planning
	• Participatory process	• Iterative, evidence based, and
		• participatory review of progress
		• Gender and equity awareness building through diversity in planning
Value and build on relationships	• Forum creation for relation between MHD – WHC – MOH	• Participatory assessment, bringing diverse stakeholders together
	• WHC as a hub for health promotion	• Mutual accountability through review of progress
	• Accountability of the municipal government to communities	
Iterative learning	• Defining assessment criteria in HICAP and WHC	• Regular measurement of outcomes in multiple dimensions of assessment
	• Involvement of MHD and WHCs in Knowledge, Practice and Coverage Household Health Surveys including disaggregation by wealth quintiles to assess equity	• Cycle of visioning, defining measures, measuring, reviewing, adjusting

^a^There is not one set of recognized systems thinking principles, and this table is not exhaustive. Williams [12] emphasizes boundaries, perspectives, and relationships as central to systems thinking. Many authors emphasize learning, and specifically iterative learning, as an essential principle. Other authors also stress the value of collaboration across disciplines, sectors and organizations, transformational leadership, alternative scenario planning, using diversity of stakeholders to brainstorm and design, etc. [6, 7, 26].

HICAP, Health Institution Capacity Assessment; MHD, Municipality Health Departments; MOH, Ministry of Health; WHC, Ward Health Committee; MOHFW: Ministry of Health and Family Welfare is the same as MOH and was removed.

**Figure 1:**
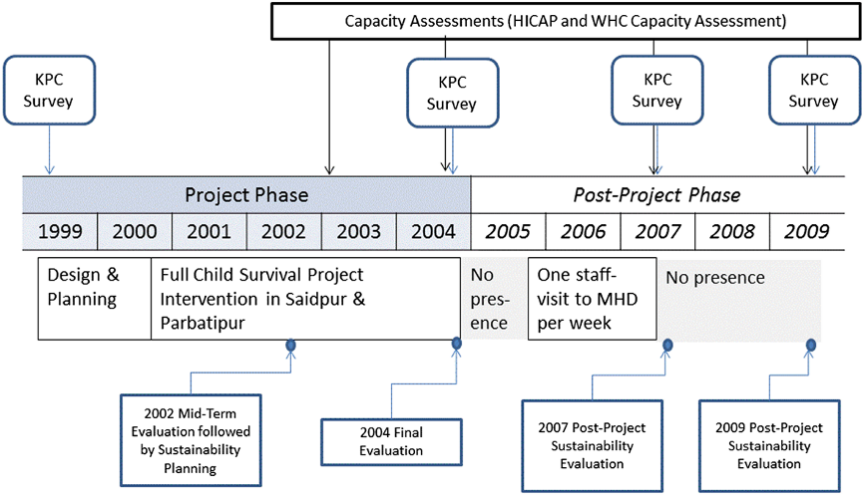
**Implementation and evaluation phases of the Saidpur and Parbatipur Child Survival Project.**

We now describe how the Sustainability Framework method was implemented following the mid-term evaluation (2002), up through the final evaluation (2004) [34], all the way to the 5-year post-project sustainability evaluation in 2009. The steps of implementation, sustainability planning, project evaluation, and post-project sustainability evaluation are summarized in Figure 1.

## Methods

The sustainability assessment is an iterative exercise designed for intervention design, evaluation, and continued learning. It requires the definition and planning of a multi-dimensional evaluation model and its major components, based on the Sustainability Framework (see “Defining sustainability for health interventions in global development” below), and measurement steps through field investigations, which took place through ongoing project monitoring and evaluation until 2004, and after the project’s end.

### Defining sustainability for health interventions in global development

We define sustainability as resulting from a collective process within a local system, which maintains or improves the health status, or a sub-set of health outcome indicators, of the locale’s citizens, particularly its most vulnerable members.

Individuals, community groups and structures, and government and civil society organizations constitute a local system within a larger environment, and it is ultimately their coordinated social interactions and efforts, based on the understanding of their own health and development, which will lead to lasting health conditions.

The loss of control over local processes beyond a set date is inherent in project approaches. This means that the immediate determinants of sustainability are based on a local process of negotiation, role definition, and action, and are effectively outside the full control of a time-bound project. Projects, nonetheless, have an essential responsibility in advancing the key determining conditions for sustaining outcomes within the local system.

**Figure 2:**
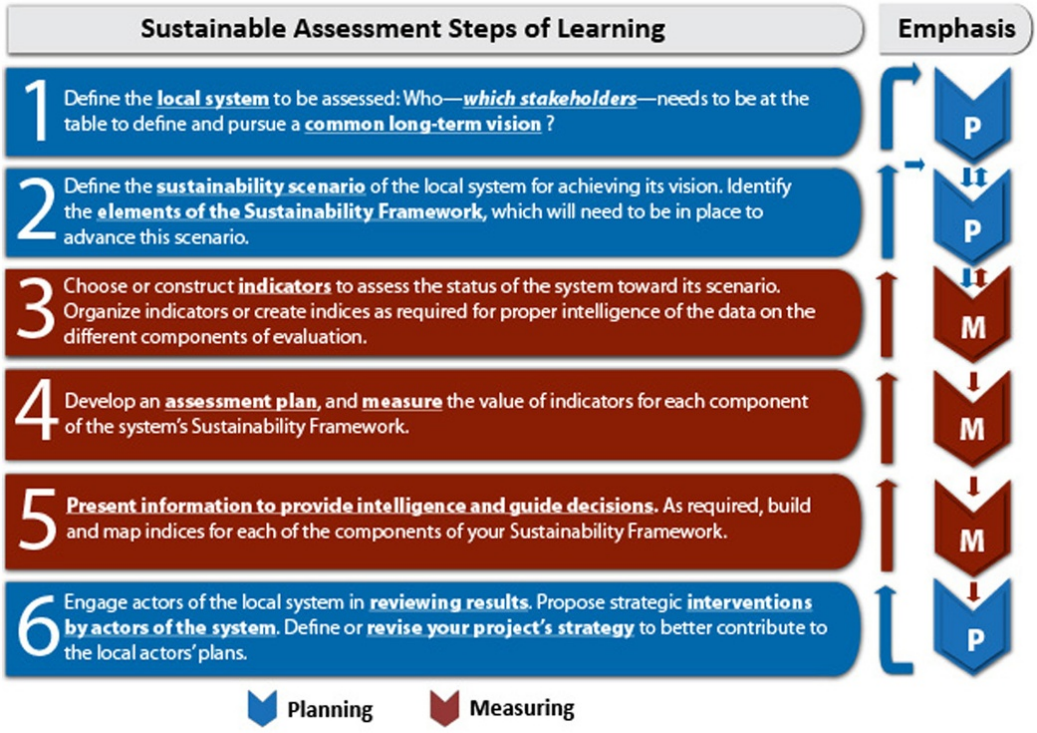
**Sustainability framework steps of learning.**

The value of the Sustainability Framework relies heavily on the quality of its contextual development and implementation process. The method is described elsewhere as a participatory process involving the six steps described in Figure 2, bringing together situation assessment, planning, evaluation, and strengthening relationships between the actors, based on consistent reference to data and learning steps [23].

We describe now how these steps were adapted by Concern and which elements of planning and evaluation were integrated in the model:

1. *Defining (mapping) the local system, and the common long-term vision*

In February 2003, Concern and its partners defined the system of local actors expected to carry out the task of health promotion at the municipality level during a 6-day workshop using stakeholder mapping, and developing a common vision through participatory group activities [14]. This initial workshop involved Concern project team members, 15 municipality staff nominated by the municipalities, and Concern capacity building and child survival advisors. All work was conducted in Bangla in small groups. Final statements were finalized in plenary and presented to municipal and ward institution leaders. This provided a safe environment for the development of a shared vision and discussion of contextual challenges. The central constituents of the system that was mapped were Mayors, elected Councilors, and the MHDs in the leadership role; WHCs as an expression of the communities, working through CHVs, and local health care providers, such as a local hospital and NGO clinics. This system definition encouraged a broader inclusion of WHC members (not just leaders) and CHV representatives in future exercises (2004, 2007, and 2009).

2) *Describe scenarios to achieve the vision, define the elements of the Sustainability Framework and their indicators (Steps 2 and 3)*

Although the language of ‘scenarios’ was not prominent at the time of Concern’s initial planning efforts, the project helped partners envision not only a workable future but also rational roles for all parties in order to ensure population health benefits were sustainable by local stakeholders. The vision and unfolding strategy were designed to integrate equity issues at the onset. Cognizant of the care-seeking barriers faced among the poorest urban dwellers, Concern sensitized elected leaders at the municipality and community levels of the importance of including all people irrespective of ethnicity or class in health promotion efforts. Leaders were encouraged to provide special assistance such as arranging transport, seeking support of an absent husband, accompanying the client to the health facility, and/or negotiating of fees and payments to those in greatest need.

The Sustainability Framework examines inter-dependent components of evaluation considered essential to sustaining health outcomes, each component including different elements. Following this, in the first step, Concern and the municipalities defined which elements of the Sustainability Framework fit their situation, and for each they defined how measurements or assessments would be carried out. Discussion and participation was maximal in defining what should be measured and which issues were of importance to achieve sustainable health outcomes. However, Concern project leaders and facilitators certainly played a leadership role in proposing indicators, and ensuring that knowledge, practice, and coverage (KPC) indicators, for example, aligned with international standards. Definition of capacity indicators and statements combined different participatory and expert influences, as described below:

- Health outcomes themselves were assessed through repeated small sample, population-based KPC health surveys [35, 36]. KPC surveys with samples of 350 to 600 mothers of children 0 to 23 months had been carried out at the onset of the project (1999) and were repeated at the end of the project (2004), and again in 2007 and 2009. Practice and coverage indicators provided the hard benchmarks to assess success or failure, as they directly reflected benefits to the population. During the process, elected leaders were challenged about inclusiveness and the participation of all community members in health promotion efforts, irrespective of ethnicity or class^b^.
- Capacity of both municipalities was assessed through the Health Institution Capacity Assessment Process (HICAP). The HICAP is a participatory, organizational self-assessment developed by Concern with staff from the municipal health departments [34]. The final selection of domains and indicators of capacity assessment was carried out by combining an appreciative inquiry approach and commonly available organizational assessment tools.
- The HICAP was then carried out with cabinet members, ward commissioners, and the health department of both municipalities in 2004, 2007, and 2009. Non-project staff at Concern led assessment workshops in Bangla, taking 3 days per municipality. The HICAP describes progress towards an “ideal capacity”, as defined by the municipality leaders through “possibility statements”. These statements provide norms of institutional behavior, as scored on a five-point scale and based on dialogue and consensus among participants.
- The Sustainability Framework considers institutional viability as related to but distinct from capacity; “*Organizational Viability, relates not only to financial viability, but also to other essential types of support and relationships—connectedness—which an organization depends on to fulfill its mission*” [16]. Through collective reflection and analysis, viability elements were identified in the model, such as Mayor-Ministry of Health collaboration and resource leveraging. Given that the project relied heavily on the leadership of the elected mayors, councilors, and their political party, the discussion of the viability of the model made clear that steps were needed to “neutralize” the political risks, and to ensure the continuity of support beyond the current municipal administrations. Local actors involved in the sustainability planning phase helped Concern take steps ranging from informing political leaders about the role of Municipalities in health promotion during the elections and promptly orienting successful candidates shortly after elections, to helping the WHCs to uphold an apolitical identity in their neighborhoods.
- The project and its partners identified the capacity of WHCs as the main proxy measure for the community. Concern developed the WHC Capacity Assessment tool, similar in structure to the HICAP, and used by the 24 Saidpur and Parbatipur WHCs in June 2004, April to June 2007, and again in 2009 to assess their own capacity. The WHC Capacity Assessment tool was informed by the HICAP development process and the national terms of reference for the WHCs. The dimensions of assessment and indicators were validated during stakeholder consultations with the municipal cabinets, health departments, and two purposively sampled WHCs in each of the municipalities. Reviews were conducted in Bangla and Urdu by trained facilitators from the project staff (in 2004) and then by municipal constituents (in 2007 and 2009). The 1-day sessions included guided discussions, followed by scoring capacity areas on a five-point scale. Areas of assessment included not only the WHC’s internal operations but also the inclusiveness of all socio-economic groups and efforts extended toward vulnerable community members and CHVs. Assessment of CHV’s coverage and retention began in 2007, but specific measures of their activities and qualities of their interventions were unfortunately not systematically monitored.
- The Sustainability Framework further challenged implementers to consider socio-economic threats that could undermine efforts towards a viable health intervention. Recurrent seasonal neighborhood flooding and cultural issues were identified as significant impediments to the desired collective vision. These impediments were addressed through providing a clear role to WHCs in coordination with emergency response and water and sanitation efforts. Additionally, provisions were made to increase social support for decision-making when a woman and/or child required immediate health care in the absence of the husband, and to generate parental and community support to allow mostly young and female CHVs to fulfill their home visit duties.

3) *Develop and implement the assessment plan (Step 4) and engage stakeholders in analysis and decision making (Steps 5 and 6)*

Both the final evaluation (2004) and the post-project sustainability assessments (2007 and 2009) involved iterative sequences of participatory evaluation steps:

- Formation of an evaluation team with Concern and municipality participants, under the guidance of an external lead evaluator.
- Analysis of surveys (health survey, capacity assessment) and available secondary data.
- Review, framing, and clarification of evaluation questions.
- Interviews of key informants, individually and through group discussions, including the mayor, cabinet members/WHC Chairs, Health Inspector, Municipal Health Staff, past and current CHVs, MOH partners, Ministry of Local Government, WHCs members, the Municipal Essential Service Package Coordinating Committee, and non-governmental health sector partners.
- Participatory review of findings, including a discussion on conclusions and next steps.

## Results and discussion

### Post-project achievements

From the moment Concern and the Municipalities took stock of mid-term achievements versus risks for sustainability, a culture of regular consultation and learning became central to the life of the project. As assessments (including the HICAP and KPC) were highly participatory, the findings of the assessments made sense to the stakeholders (as suggested by the actions taken by stakeholders, and discussions with the evaluator). Participation in the selection of indicators was, of course, informed by population surveys specialists and technical guidance. However, the role of local stakeholders went beyond obtaining authorizations. Municipal health departments were part of the design team defining which indicators should be measured, and were then central to the analysis and discussion of the results.

The overall findings of the 2009 post-project sustainability evaluation was largely positive: “*From 2004 (end of the project) to 2007 (first post-project sustainability evaluation), in spite of a near total [98%] reduction of external inputs, the municipalities were able to maintain basic operations and observed mostly stable values for maternal and child health outcomes. From 2007 to 2009 (last post-project sustainability evaluation), in the absence of any further inputs by Concern, basic capacity, operations, and health indicators were maintained, but municipalities identified critical gaps in the governance and strategic guidance of the model, despite weaknesses in human resources management and national level involvement*” [37].

Table 2 presents the evolution of 11 indicators compared to national urban trends during and after the project. We can summarize the table as changes observed during and after the life of the project:

**Table 2:** **Child Health Indicator Trends in Saidpur and Parbatipur (KPC) and Bangladesh Demographic and Health Survey Comparisons (urban or national average)**

	Indicator		1999	2004	1999–2004 diff (***P***value)	2007	2009	2004–2009 diff (***P***value)
Child Health	Complete immunization	S&P*	44%	91%	<0.05	90%	91%	NS
		urban	70%	81%		86%		
	Vitamin A supplementation	S&P	37%	78%	<0.05	71%	79%	NS
		urban	76%	85%		90%		
	Exclusive breastfeeding	S&P	55%	72%	<0.05	70%	73%	NS
		nat’l	42%	36%		43%		
	Complementary feeding of children 6 to 11 months	S&P	46%	64%	<0.05	64%	65%	NS
		nat’l						
	Additional feeding and fluids for the sick child	S&P	25%	44%	<0.05	34%	25%	<0.05
	Additional fluids (only) for the sick child	nat’l	50%	52%		38%		
	Proper child acute respiratory infection identification and referral	S&P	24%	34%	<0.05	66%	52%	<0.05
		urban	48%	35%		49%		
Maternal & Neonatal Health	At least one prenatal consultation during last pregnancy	S&P	59%	89%	<0.05	87%	95%	NS
		nat’l	37%	56%		60%		
	At least three prenatal consultation during last pregnancy	S&P		64%		62%	74%	<0.05
		nat’l	25%	27%		32%		
	At least one tetanus toxoid dose during last pregnancy	S&P	46%	89%	<0.05	57%	70%	<0.05
		urban	88%	88%		84%		
	Delivery by skilled attendant	S&P	31%	50%	<0.05	56%	59%	<0.05
		nat’l	22%	13%		18%		
	Delivery in health care facility	S&P	25%	45%	<0.05	49%	57%	<0.05
		nat’l	8%	9%		15%		
	Immediate breastfeeding	S&P	26%	57%	<0.05	50%	64%	NS
		urban	23%	22%		41%		

Source: S&P (Saidpur and Parbatipur), knowledge, practice and coverage (KPC) surveys 1999, 2004, 2007, 2009; national or urban estimates: Bangladesh Demographic and Health Survey.

- These 11 coverage indicators showed notable improvements during the life of the project. The 2004 final evaluation reviewed possible confounding factors to the attribution of results to the project and was supportive of a substantial attribution of effect to the project [34]. Impact on equity was only taken into account by Concern in a follow-on project in seven municipalities. However, coverage indicators for Saidpur and Parbatipur in 2004 (end of project) among the poorest 20% of surveyed households were two to five times that of the 2005 baseline estimates in the seven neighboring municipalities. Given the relative comparability of the initial and expansion municipalities, this could suggest that impact in Saidpur and Parbatipur had been far from negligible among its poorest population.
- By the time of the post-project sustainability evaluations (2007 and 2009), the main point of the evaluation shifted from the project to providing information to the municipalities themselves about progress toward their vision of sustainable health. Questions of attribution of results to the original project itself became less critical. As shown in Table 2, the initial improvements in 11 indicators of maternal and child health realized during the project (1999 to 2004), 9 were maintained or improved during the 5 years post-project, even though external funding dropped to almost zero over this period^c^. In only two cases did an indicator worsen between 2004 and 2009.

Self-assessment of capacity at the MHDs, using the HICAP tool, progressed substantially from the 2002 to 2003 baseline assessments to the end of the project (2004). By 2007, the scores on the HICAP faced a ceiling effect. The assessment had, however, helped guide and institutionalize the basic functions and operations required of the MHDs to support health promotion in the community, including through small amounts of financial support to each WHC.

The 2009 assessment revealed maintenance of the structure and basic functions of the WHCs, along with weaknesses in their operations. All WHCs had maintained a bank account with a solid balance. They mobilized additional resources and obtained financial support from the municipalities for special events, as well as emergencies affecting the poorest members of the community. WHCs, nonetheless, expressed dissatisfaction with the inconsistent support from municipalities.

While performance issues at the WHC level and in the WHC support of CHVs were identified, the human infrastructure continued to operate at the time of the 5 year post-project sustainability assessment (2009), despite a high degree of national political instability and the food price crisis of 2007 to 2008. The diversification of perspectives from diverse actors through the sustainability assessment phase allowed avoiding the natural bias of representation (male, elite) within the WHC and led WHC membership to be more representative of all segments of the neighborhood (i.e., class, ethnicity, education level, gender, and political affiliation). Additionally, having formed a vision of a desirable public good with a diverse group of stakeholders, the project was able to involve them in informing political party leaderships (both in power and opposing) about the role of WHCs and the Councilors. By the time of the post-project sustainability assessment, most WHCs had gone through a change of Chairpersons following municipal elections, but continued operating as largely apolitical institutions dedicated to promoting the common good.

### Sustainability needs to be assessed as resulting from a local system’s process, not an end-point

Concern worked with stakeholders and generated a process-within-a-system. The level of sustainability achieved was a partial achievement. It proceeded from also partial but expressed capacity, collaboration, coordination, occasional cooptation, some loss of energy but maintenance of key elements, such as operations of the WHCs and CHVs. While efforts of different parties may have been imperfect, they aimed to contribute to a recognized public good (preventive health outcomes). Essentially, sustainability occurred as a process supported by a network of system agents^d^.

Concern clearly encouraged this by stepping out of direct implementation and very rapidly supporting local stakeholders in negotiating their long term roles. The project not only aligned to a national policy, but since it had not been implemented on the ground, it operationalized it and helped local stakeholders in giving it substance.

The fact that both mayors and most elected officials participated in the sustainability assessment 5 years after the end of activities speaks of the ownership that was created. This was built through very intense and persistent efforts at “accompanying”, or softly leading councilors, staff, WHC members, and volunteers in the early phases of the project.

Interestingly, the approach of Concern, supported by a planning and evaluation tool for sustainability, can be compared to systems thinking design steps highlighted in the Alliance for Health Policy and System Research’s “flagship publication” [6]: 1) The project convened stakeholders repeatedly and at every step. 2) Concern led not only collective brainstorming but also helped stakeholders define the road ahead, the modes of monitoring and evaluation, down to the indicators when possible, and then jointly review findings (not just with the leaders and experts). 3) The definition of elements of the Sustainability Framework provided a conceptual map of expected results and scenarios for progress. 4) The iterative steps of assessment, action, and reviews allowed a measure of adaptation at each step. The last post-project sustainability evaluation showed, to some extent, how this assessment for action principle had been institutionalized by the municipality partners. While Concern had stopped all involvement in the municipality for some years at this point, apart from the evaluation itself, stakeholders largely self-organized and decided to work into the evening to define their response to the findings of the sustainability evaluation.

### The sustainability framework did not offer a perfect measurement tool, but served the role of a local systems thinking and learning tool

The ongoing learning through the project phases of implementation and assessment took place despite some elements of the Sustainability Framework lacking measures. For example, indicators of community capacity focused on community-based organizations (WHCs) but failed to capture larger social processes and social capital formation, probably elements of equal if not greater import. The literature suggests that this remains a challenge especially outside of research programs [38].

Some components of the Sustainability Framework are more amenable to standard, reliable measures than other components, assessed through softer methods. It is the resulting combined evidence which allowed actors of the system to engage in systems thinking and making sense of the data:

- Proxy measures of health outcomes are well codified through both demographic and health surveys [27–29] and small population surveys [35], such as the KPC Survey used by Concern [36].
- The HICAP results were initially extremely useful in identifying structural weaknesses in the urban health institutional infrastructure. The heavy investment of the municipalities in refining and adapting the tool, while it did not allow for standardization, provided strong buy-in and critical reflection from participants on their collective capacity, even if the benefit of the tool waned by 2009 (due to ceiling effects and lack of new information provided by the tool).
- The consideration of viability and social-economic environment forced a wider perspective in analyzing how the external health intervention plays out in the development context. The tool required the project to consider inter-relationships between municipal authorities, the Ministries of Health, and civil society. The process assisted stakeholders in vetting risks to the health promotion efforts and give them higher priority in their efforts than they would have otherwise. For example, flood mitigation measures and female mobility for both CHVs and health referrals became central tenants of the community mobilization strategy, even if they had not been explicit mandates of the original project.

The Sustainability Framework provided a tool and a method to engage stakeholders in learning evidence-based steps. Two features of the tool proved useful:

- Being systematic is a content issue: the Sustainability Framework helped users to consider distinct dimensions of progress systematically, each with defined content elements and corresponding measures. The identification of the components of the model had strong face validity and remained meaningful to local stakeholders throughout the process.
- Being systemic is a process issue: the Sustainability Framework considered a local system and, before focusing on measures of capacity and performance, sought to understand the relationships, both existing and those to be negotiated, between the members of the system.

The process for fleshing out the content of the planning or evaluation model is one which requires connections and interplay between diverse and interdependent entities in the system (i.e., the WHCs and the MHD). In so doing, stakeholders also are encouraged to interact and, if using the iterative process, over time, build some common language, trust, and goals (social capital) [39].

A crucial step in this process was the development of a common vision by all stakeholders and the continuous reference to the shared mandate it created. A system of local actors possibly lacking coherence, common currency, trust, and positive experience of joint achievements will be limited in the vision it can frame. In Saidpur and Parabatipur, this process was facilitated greatly by Concern at the onset. By the 5-year post-project period, key elements of that vision were still shared and alive (the existence and importance of WHCs and volunteers, the need to support the most vulnerable citizens from the worst shocks). The fact that the local vision built on the realization of a national policy certainly helped local actors to define and embrace it initially; but by the time of the final study, it was maintained not by the will of a fairly absent central government, but by its meaning to local actors.

### Ownership is inherently challenged by external assistance; early consideration of sustainability and a systems approach to sustainability evaluation can mitigate this risk

An inherent tension has existed between the concepts of external assistance and local ownership over the course of the last 70 years of development assistance. The Sustainability Framework does not pretend to offer the solution to this quandary, but it does support a local system of actors exploring different visions and possibilities for a more successful pursuit of a common goal. It also helps external actors become change agents of and contributors to this system. It offers a way to reduce the displacement of ownership, which money naturally brings to resource-constrained environments.

The initial focus on defining the system, bringing different groups to the table, and trying to build a common vision and compatible scenarios are possibly the most important learning steps advanced by the Sustainability Framework, along with regular monitoring and reviews. Through iteration of measures, negotiation and decision making, the process required by the Sustainability Framework can help the local system adapt to successes, to new events, and shocks. Sustainability means that the system is able to conceive of, then realistically adapt and develop new ideas, thus transforming or evolving the scenario its members initially imagined.

Saidpur and Parbatipur clearly reached a stage where new options were conceivable to them through an institutional infrastructure which reached their most vulnerable members. The sustainability assessment identified choices, which municipalities had to make to seize on these options.

## Conclusions

The challenge for Concern was to provide an ongoing evaluation process which would be evidence-based, allow effective implementation of interventions by municipal structures, and inform the social and health system actors about progress toward sustainable health achievements.

The process of joint visioning, planning, implementation, monitoring, assessment, review, and decision-making provided Saidpur and Parbatipur with a systematic, if perfectible, approach to do this. The answer made sense to the local actors from communities and municipalities, including health officials who participated in the sustainability evaluation. In the end, the Sustainability Framework played maybe its most important role as a tool for engagement of and negotiation between local stakeholders. It offered a guide to self-learning and decision-making with an evidence-based focus on objectives and tangible public good (health indicators, WHCs, and CHV activities).

Social, political, and organizational systems have the particularity of being purposeful complex adaptive systems^d^ [40], which means that agents are endowed with some level of free will to define their own individual strategies within a system, based on information received about other agents’ behaviors. A systems approach, operationalized through the Sustainability Framework, reduced the tension in balancing sustainability and equity. As all key stakeholders were present and engaged in the framing of a vision inclusive of equitable concerns, the development of action plans and metrics of success had to include prioritization of the neediest. Furthermore, the constant reference to data, metrics of progress understood by all, referring back to a long-term vision repeated with constancy and visualized in assessment reports, allowed the construction of what Geyer and Rihani call a “societal framework” through which the value of the public good being pursued is reinforced for all [41].

Development aid’s efforts at scale up and acceleration of achievements are known to create stress on country systems, regardless of good intentions. This makes the question of sustainability still enormously critical to the future of global health and global development [42, 43]. Development projects too often deal with sustainability as a false promise or a utopia, with statements such as, “*the project will ensure sustainability three years from now by…*”; this does not lend itself to shared accountability for progress on an authentic process worth the efforts of beneficiaries, country stakeholders, project designers, implementers, and donors. Commitment to sustainability requires us to approach more honestly and rigorously its unfortunate complexity. And as illustrated in the section “A systems understanding of sustainability,” we are still learning to recombine or create tools to effectively use “systems thinking” on complex issues such as ownership, scale, and sustainability [43–45]. We hope to have illustrated the value of one such approach.

Finally, given the evidence for the challenging conditions under which sustainability can develop at local levels and the time this requires, national governments themselves, with or without foreign aid, will benefit from more methodical and systems-oriented planning evaluation methods to a complex but essential question.

## Endnotes

^a^http://www.coregroup.org/about-us/history-of-core-group

^b^In a follow-on project in new municipalities, Concern added a module to the external baseline and final household surveys, allowing comparison of health outcomes across proxy wealth quintiles [46].

^c^With the exception of a USAID-supported project promoting facility deliveries, during the life and after the end of the project.

^d^A discussion of complex adaptive systems behaviors of both municipalities and project is available [47].

## Abbreviations

CHVs: Community health volunteers
Concern: Concern Worldwide Inc.
HICAP: Health Institution Capacity Assessment Process
KPC: Knowledge, practice, and coverage
MHDs: Municipal health departments
NGOs: Non-Governmental Organizations
USAID: US Agency for International Development
WHCs: Ward Health Committees.
